# Intramyocellular lipid accumulation after sprint interval and moderate‐intensity continuous training in healthy and diabetic subjects

**DOI:** 10.14814/phy2.13980

**Published:** 2019-02-10

**Authors:** Tanja Sjöros, Virva Saunavaara, Eliisa Löyttyniemi, Mikko Koivumäki, Ilkka H. A. Heinonen, Jari‐Joonas Eskelinen, Kirsi A. Virtanen, Jarna C. Hannukainen, Kari K. Kalliokoski

**Affiliations:** ^1^ Turku PET Centre University of Turku Turku Finland; ^2^ Turku PET Centre Turku University Hospital Turku Finland; ^3^ Department of Medical Physics Division of Medical Imaging Turku University Hospital Turku Finland; ^4^ Department of Biostatistics University of Turku Turku Finland

**Keywords:** Exercise, insulin sensitivity, muscle, proton magnetic resonance spectroscopy, sprint interval training, triacylglycerol

## Abstract

The effects of sprint interval training (SIT) on intramyocellular (IMCL) and extramyocellular (EMCL) lipid accumulation are unclear. We tested the effects of SIT and moderate‐intensity continuous training (MICT) on IMCL and EMCL accumulation in a randomized controlled setting in two different study populations; healthy untrained men (*n* 28) and subjects with type 2 diabetes (T2D) or prediabetes (*n* 26). Proton magnetic resonance spectroscopy (^1^H MRS) was used to determine IMCL and EMCL in the Tibialis anterior muscle (TA) before and after a 2‐week exercise period. The exercise period comprised six sessions of SIT or MICT cycling on a cycle ergometer. IMCL increased after SIT compared to MICT (*P* = 0.042) in both healthy and T2D/prediabetic subjects. On EMCL the training intervention had no significant effect. In conclusion, IMCL serves as an important energy depot during exercise and can be extended by high intensity exercise. The effects of high intensity interval exercise on IMCL seem to be similar regardless of insulin sensitivity or the presence of T2D.

## Introduction

It is well‐known that aerobic exercise has many health‐enhancing effects. Sprint interval training (SIT) has been shown to be at least as effective as moderate‐intensity continuous exercise in inducing health benefits (Sloth et al. 2013; Gist et al. 2014). However, the effects of SIT on intramyocellular lipid (IMCL) accumulation remain unclear. The lipid droplet size in the subsarcolemmal space has decreased after high‐intensity interval training (HIIT) compared to endurance training measured by electron microscopy, but no change in the total lipid volume within the myocytes has been observed (Koh et al. 2018).

During aerobic exercise, lipids are used as energy substrates, resulting in decreased IMCL stores (Egger et al. 2013; Bucher et al. 2014). The depleted lipid stores are replenished during the next 24 h and hypercompensated in 30–70 h after exercise by sufficient nutritional fat supply (Décombaz et al. 2001; Larson‐Meyer et al. 2002). Thus, regular exercise training can, in the long term, lead to increased IMCL in both healthy and insulin resistant subjects (Tarnopolsky et al. 2007; Haus et al. 2011). It is evident that IMCL stores respond rapidly to changes in physical activity and diet. Abundant IMCL has been associated with whole‐body and central adiposity (Ingram et al. 2011; de la Maza et al. 2015) and insulin resistance (Goodpaster et al. 1997; Krssak et al. 1999), as well as with aerobic capacity (Thamer et al. 2003). Paradoxically, both insulin resistant subjects and endurance athletes have high amounts of IMCL (Goodpaster et al. 1997, 2001; Krssak et al. 1999; van Loon et al. 2004). Extramyocellular lipid (EMCL) stores are located between the myocytes but within the macrostructure of the muscle, and their role in muscle metabolism remain unresolved.

As a part of a larger research project we assessed the effects of SIT and moderate‐intensity continuous training (MICT) on IMCL accumulation in two different study populations; healthy untrained men and subjects with type 2 diabetes (T2D) or prediabetes. We used proton magnetic resonance spectroscopy (^1^H MRS) to determine the IMCL and EMCL in the tibialis anterior muscle (TA) before and after a 2‐week exercise intervention of SIT or MICT cycling. Previously we showed that, in the same study subjects, after 2 weeks of SIT or MICT, whole‐body and skeletal muscle insulin sensitivity was increased both in healthy sedentary men (Eskelinen et al. 2015) and individuals with T2D or prediabetes (Sjöros et al. 2018). Additionally, skeletal muscle free fatty acid uptake (FAU) was increased after exercise in the Quadriceps femoris muscle (QF) in the group of diabetic subjects (Sjöros et al. 2018), but not in the group of healthy men (Eskelinen et al. 2015). Therefore, we hypothesized that after 2 weeks of SIT or MICT, IMCL would increase in the group of subjects with impaired glucose metabolism but remain unaltered in the group of healthy sedentary men. Furthermore, maximal oxygen consumption in a maximal exercise test (VO_2peak_) increased only after SIT exercise in the group of diabetic or prediabetic subjects (Sjöros et al. 2018), therefore we hypothesized that SIT and MICT training would also have different effects on IMCL.

## Materials and Methods

This study was a parallel‐group randomized controlled trial. The study was conducted at the Turku PET Centre, Turku, Finland between March 2011 and October 2015. All measurements were done at Turku PET Centre except for the VO_2peak_ tests and body composition measurements, which were performed at the Paavo Nurmi Centre, University of Turku, Turku, Finland.

The study was performed according to the Declaration of Helsinki and was approved by the Ethics Committee of the Hospital District of South‐Western Finland (decision 95/180/2010 §228). The present study is a part of a larger study titled: ‘‘The Effects of Short‐Term High‐Intensity Interval Training on Tissue Glucose and Fat Metabolism in Healthy Subjects and in Patients with Type 2 Diabetes’’ (NCT01344928).

## Subjects

The subjects were recruited in South‐Western Finland in two phases as previously described (Kiviniemi et al. 2014; Sjöros et al. 2018). In the first phase, 28 previously untrained [age 48 (SD 5) years] healthy men were recruited. In the second phase, 26 untrained [age 49 (SD 4) years], individuals (10 women) with either non‐insulin‐treated T2D or prediabetes (impaired glucose tolerance, IGT and/or impaired fasting glucose, IFG) were admitted. The inclusion criteria were as follows: age of 40–55 years and a good treatment balance in case of T2D. The exclusion criteria were as follows: any other chronic disease or defect which hinder daily life, smoking or use of narcotics, history of anorexia nervosa or bulimia, history of asthma, current or history of regular and systematic exercise training, VO_2peak_ > 40 mL·kg^−1^·min^−1^, or any other condition that in the opinion of the investigator could create a hazard to the participant's safety, endanger the study procedures, or interfere with the interpretation of the study results (Eskelinen et al. 2015; Sjöros et al. 2018). Seventeen of the subjects in the second group met the criteria of T2D, seven had both IGT and IFG, and two had IFG. Nine of the subjects in the SIT group and four in the MICT group were treated by oral hypoglycemic agents (Sjöros et al. 2018) (Table 1). The subjects were instructed not to alter their dietary habits or daily activities during the study. After all pre‐intervention measurements the subjects were randomized by random permuted blocks into SIT or MICT training group. The observer analyzing the MRS data (V.S.) was blinded to the group allocation.

**Table 1:** Numbers of study subjects and medical treatments in each group

	T2D/prediabetes		Healthy	
	SIT	MICT	SIT	MICT
*n*	13	13	14	14
Men/women, *n*	9/4	7/6	14/0	14/0
DG, *n* (T2D)/*n* (IGT/IGF)	11/2	6/7	0/0	0/0
Glucose lowering medication, *n*				
Metformin	7	4	0	0
DPP‐4 inhibitors (sitagliptin)	4	1	0	0
Sulfonylurea (glimepiride)	1	0	0	0
Other medication, *n*				
Antihypertensives	5	6	0	0
Statins	4	3	0	0
Affective medication	0	3	0	0
Menopausal hormone therapy	1	2	0	0

### Magnetic resonance measurements

**Figure 1:**
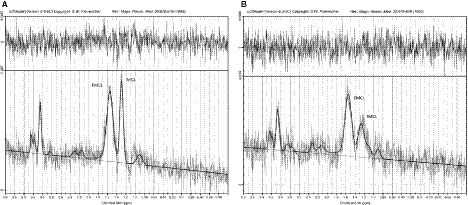
Spectra of TA muscle of one study subject recorded at 1.5 T measured (A) before and (B) after the intervention. Images show the measured data and the fit (black line) and the residuals at the top. The residuals are randomly scattered. Data for IMCL (1.3 ppm) and EMCL (1.5 ppm) methylene protons were used for statistical analyses.

IMCL and EMCL in the TA were measured by proton magnetic resonance spectroscopy (^1^H MRS) using a Philips 1.5T Gyroscan Intera CV Nova Dual MR scanner (Philips Medical Systems, Best, The Netherlands). A surface coil, E1 coil (Philips Medical Systems, Best, The Netherlands), was used in the measurement. Spectra were obtained from a single 18 mm × 10 mm × 10 mm voxel within the TA. The voxel was placed in the muscle on anatomical transverse, sagittal and coronal images of the lower leg avoiding vascular structures and visible fat. Images were taken using fast field echo (FFE) sequence. MRS was done using point resolved spectroscopy (PRESS) sequence. An echo time of 27 msec and repetition time of 3000 msec were used and 128 averages. Data were analyzed using the linear combination of model spectra software package (LCModel) version 6.3‐0C (Provencher 1993) (Fig. 1). Both eddy‐current correction and water scaling were used in the analysis. The measurements were done in the fasting state at least a week after the VO_2peak_ test before the intervention and at least 48 h after the intervention.

### Other measurements

The whole‐body insulin‐stimulated glucose uptake rate was measured by a hyperinsulinemic euglycemic clamp as previously described on the following day of the MRS measurements before and after the intervention (Eskelinen et al. 2015; Sjöros et al. 2018). A primed‐constant insulin (Actrapid, 100 U·mL^−1^, Novo Nordisk, Bagsvaerd, Denmark) infusion was started with the rate of 40 mU·m^2^ of body surface area in a minute during the first 4 min. From 4 to 7 min, infusion rate was reduced to 20 mU·m^−2^·min^−1^, and, from 7 min to the end of the clamp, it was kept constant at 10 mU·m^−2^·min^−1^. Exogenous glucose infusion was started 4 min after the start of the insulin infusion with a rate of subject's weight (kg)·0.1^−1^·g^−1^·h^−1^. At 10 min, glucose infusion was doubled, and after that further adjusted according to blood glucose concentration to keep it as closely as possible to the level of 5 mmol·L^−1^. Arterialized venous blood samples were collected every 5–10 min to determine the glucose concentration for adjusting the glucose infusion rate (Eskelinen et al. 2015).

VO_2peak_ tests were performed as previously reported (Kiviniemi et al. 2014). The test was started at 50 W and followed by an increase in 30 W every 2 min until volitional exhaustion. Ventilation and gas exchange (Jaeger Oxycon Pro; VIASYS Healthcare) were measured and reported as the mean value per minute. The highest 1‐min mean value of oxygen consumption was used as VO_2peak_ (Kiviniemi et al. 2014). Body fat percentage was measured using a bioimpedance monitor (InBody 720, Mega Electronics Ltd., Kuopio, Finland).

Free fatty acid uptake (FAU) in thigh and upper arm muscles (quadriceps femoris, hamstrings, biceps and triceps brachii) was measured with positron emission tomography and [^18^F]FTHA tracer as previously described (Eskelinen et al. 2015). The tracer was injected into the antecubital vein and scanning of the thoracic region (with biceps and triceps brachii muscles) was started simultaneously. The femoral region (quadriceps femoris and hamstring muscle groups) was scanned starting approximately at 65 min after the tracer injection in 3 × 300 sec time frames. Starting at 4 min after the tracer injection, blood samples for plasma radioactivity determination (Wizard 1480 3; Wallac, Turku, Finland) and calculation of input function were collected at approximately (exact timing was recorded) 4, 5, 7.5, 10, 20, 30, 55, and 70 min time points. Additional blood samples were collected to measure the metabolites of the [^18^F]FTHA to make pure plasma [^18^F]FTHA input function (Eskelinen et al. 2015). Mean skeletal muscle FAU was calculated as the mean of the above‐mentioned measured muscles. Plasma FFA concentration was measured at the laboratory of Turku University Hospital using standard assays.

### Training interventions

Both SIT and MICT groups performed six supervised exercise sessions during 2 weeks as previously described (Heiskanen et al. 2016; Sjöros et al. 2018). One SIT session comprised 4–6 all‐out 30 sec cycling intervals with 4‐min recovery periods (Monark Ergomedic 894E, Monark, Vansbro, Sweden). A MICT session comprised 40–60 min of cycling at 60% of peak workload (Tunturi E85, Tunturi Fitness, Almere, The Netherlands).

### Statistical methods

All the subjects with successfully measured MRS data obtained both before and after the intervention or at either of the two time points were included in this analysis population. The MR measurements were successfully completed for 49 subjects before and for 43 subjects after the intervention. From the acquired data, the IMCL and EMCL peaks could not be distinguished in every case, leading to 48 and 42 IMCL results and 44 and 38 EMCL results before and after the intervention, respectively. One case of extreme outlier value in IMCL after the intervention and two cases of extreme outlier values in EMCL after the intervention were further excluded from the analysis to ensure normal distribution.

The normal distribution of the data was evaluated visually and using the Shapiro‐Wilks test. The square root transformations for the IMCL, EMCL, fasting FFA and FAU in the hamstrings were made for the analyses to obtain normal distribution of the data. Statistical analyses were performed using a hierarchical mixed linear model with a compound symmetry covariance structure, including one within‐factor (time), two between‐factors [diabetes status (healthy and T2D/prediabetic); training group (SIT and MICT)] and interaction terms time*diabetes(sex) and time*training. Diabetes factor (healthy or T2D/prediabetes) was nested to sex since we only had men in the group of healthy subjects. Missing data points were accounted for by restricted maximum likelihood estimation within the model. We report model‐based means and their 95% confidence intervals for all the parameters. All statistical tests were performed as two‐sided with statistical significance level set at 0.05. Pairwise correlations between the parameters in pre‐ and post‐training situations and in changes of parameters over time were calculated using Pearson's correlation coefficient. For non‐normally distributed data Spearman's rank correlation coefficient was used. The analyses were performed using SAS System, version 9.3 for Windows (SAS Institute, Cary, NC).

## Results

At the baseline, no statistically significant differences were found between the training groups (in the parameters presented in Table 2). Healthy and diabetic/prediabetic subjects differed in BMI and body fat percent, in blood glucose and insulin levels as well as in VO_2peak_, glucose tolerance, insulin sensitivity, and muscle FAU (Table 2).

**Table 2:** Study subject characteristics at the baseline in the SIT and MICT exercise groups

	SIT			MICT			*P*		
	Healthy men	Diabetic men	Diabetic women	Healthy men	Diabetic men	Diabetic women	Group	Diab (sex)	Group × diab (sex)
Age, years	47.4 (45.1; 49.6)	47.3 (44.5; 50.2)	53.2 (48.9; 57.4)	47.9 (45.6; 50.2)	47.3 (44.1; 50.5)	49.7 (45.9;53.5)	0.46	0.051	0.47
BMI, kg·m^−2^	25.9 (24.4; 27.3)	30.4 (28.6; 32.2)	30.1 (27.4; 32.8)	26.4 (24.9; 27.8)	31.1 (29.1; 33.2)	30.9 (28.5; 33.4)	0.42	<0.001	0.99
HbA1c, mmol·L^−1^	36.5 (34.1; 38.8)	39.1 (36.1; 42)	40.7 (36.3; 45.1)	37.4 (35; 39.8)	40.2 (36.9; 43.5)	38.7 (34.8; 42.7)	0.98	0.088	0.66
HbA1c %	5.5 (5.3; 5.7)	5.7 (5.5; 6)	5.9 (5.5; 6.3)	5.6 (5.4; 5.8)	5.8 (5.5; 6.1)	5.7 (5.3; 6.1)	0.98	0.094	0.67
Fasting glucose, mmol·L^−1^	5.4 (5; 5.8)	7.5 (7.1; 8)	7 (6.3; 7.7)	5.6 (5.2; 6)	7 (6.4; 7.5)	6.6 (6; 7.3)	0.25	<0.001	0.23
OGTT 2 h glucose, mmol·L^−1^	5.4 (4.2; 6.6)	11.5 (10.1; 13)	10.5 (8.3; 12.6)	6.2 (5.1; 7.4)	10.7 (9.1; 12.3)	10.7 (8.8; 12.6)	0.93	<0.001	0.46
Fasting insulin, pmol·L^−1^	5.2 (1.1; 9.3)	15.6 (10.6; 20.5)	11.5 (4.1; 18.9)	5.6 (1.7; 9.5)	20 (14.4; 25.6)	12.8 (6.2; 19.4)	0.37	<0.001	0.68
OGTT 2 h insulin, pmol·L^−1^	23.8 (0.6; 47.1)	91.4 (63.5; 119.4)	75.8 (33.9; 117.7)	39.5 (17.1; 61.9)	79 (47.3; 110.7)	70.6 (33.1; 108.1)	0.96	<0.001	0.54
VO_2peak_, mL·kg^−1^·min^−1^	34.7 (32.7; 36.8)	28 (25.2; 30.7)	25.2 (21.3; 29.1)	33.7 (31.6; 35.8)	30.9 (27.9; 33.8)	24 (20.1; 27.9)	0.87	<0.001	0.25
*M*‐value, μmol·kg^−1^·min^−1^	38.2 (31.5; 44.9)	16.1 (6.6; 25.6)	25.8 (13.2; 38.3)	31.9 (24.7; 39.1)	14.9 (4.7; 25.2)	12 (0.8; 23.2)	0.08	<0.001	0.51
Fat %	22.2 (19.8; 24.7)	29.3 (26; 32.5)	40.5 (35.9; 45.1)	22.9 (20.4; 25.3)	28.4 (24.9; 31.8)	40.5 (35.9; 45.1)	0.96	<0.001	0.86
Mean FAU, μmol·100 g^−1^·min^−1^	4.6 (3.8; 5.4)	3.2 (2.2; 4.1)	4.5 (2.8; 6.1)	5.5 (4.7; 6.3)	3.4 (2.3; 4.4)	5 (3.7; 6.3)	0.25	0.001	0.74

The results are presented as model‐based means (95% confidence interval). Group *P*‐value indicates whether there is a difference between the exercise groups; diab(sex) *P*‐value indicates whether there is a difference between healthy and diabetic or prediabetic subjects. Diabetes factor (healthy or T2D/prediabetes) was nested to sex since we only had men in the group of healthy subjects.

SIT, sprint interval training; MICT, moderate intensity continuous training; BMI, body mass index; HbA1c, glycated hemoglobin; OGTT, oral glucose tolerance test; VO_2peak_, maximal oxygen uptake in a maximal exercise test; *M*‐value, the whole‐body insulin‐stimulated glucose uptake rate under hyperinsulinemic euglycemic clamp; FAU, skeletal muscle fatty acid uptake measured by positron emission tomography.

During the intervention, three participants in the SIT group dropped out, one due to training‐induced hip pain, one due to migraine headache during the first exercise session, and one due to claustrophobic sensation in the MR scanner. Four participants in the MICT group dropped out due to personal reasons. The compliance to the exercise protocol was good. All the subjects who completed the post‐intervention measurements also completed all the exercise sessions.

### Training effects

**Figure 2:**
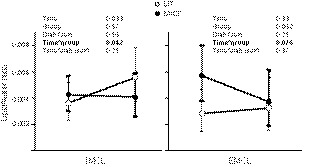
Water to lipid ratio of IMCL and EMCL in the tibialis anterior muscle measured by proton magnetic resonance spectroscopy. Open dots show the results of the SIT group and black dots the results of the MICT group before and after the intervention presented as model‐based means with 95% confidence interval. Mean FAU in the skeletal muscles, body fat percentage, insulin sensitivity (*M*‐value in the hyperinsulinemic euglycemic clamp), and VO_2peak_ are included as factors in the model. Time *P*‐value displays the mean change between pre‐ and post‐measurements; group *P*‐value indicates whether there is a difference between the exercise groups; diab(sex) *P*‐value indicates whether there is a difference between the results of healthy and diabetic or prediabetic subjects; time*group *P*‐value indicates whether the mean changes over time are different between exercise groups; time*diab(sex) *P*‐value indicates whether the mean changes over time are different between healthy and diabetic or prediabetic subjects.

In the primary analysis, the IMCL in TA in the patients with T2D or prediabetes was statistically significantly higher (*P* = 0.0012) compared to healthy subjects. There were no significant differences between the training effects among subject groups (healthy or T2D/prediabetes) or training modes (SIT/MICT). There was a significant overall training effect (*P* = 0.028), indicating a similar increase in IMCL in both subject groups by both SIT and MICT training modes. When mean FAU in the skeletal muscles, body fat percentage, insulin sensitivity (*M*‐value in the hyperinsulinemic euglycemic clamp) and VO_2peak_ were added as factors into the model, the overall training effect (time) was no longer significant (*P* = 0.10) and the difference between subject groups (healthy or T2D/prediabetes) disappeared (*P* = 0.16). In this model, the time*training interaction was significant (*P* = 0.042), which indicates a more pronounced increase in IMCL by SIT compared to MICT (Fig. 2).

On EMCL, the training intervention had no effect in the primary analysis (*P* = 0.12 for the time effect). There were no differences between subject groups or in the training effects between groups (*P* ≥ 0.17 in all comparisons). The results remained similar when mean FAU, fat percentage, *M*‐value and VO_2peak_ were added as factors into the model. Fat percentage had a significant effect in the model (*P* = 0.028). There was a tendency (*P* = 0.076) towards time*group interaction: EMCL tended to decrease in the MICT group while it remained unaltered in the SIT group (Fig. 2).

### Correlations

No simple correlations between the change in IMCL and changes in body adiposity, tissue insulin sensitivity, or aerobic capacity were found. At the baseline, a negative correlation between insulin sensitivity (*M*‐value in the hyperinsulinemic euglycemic clamp) and IMCL was found (*r* = −‐0.42, *P* = 0.005). However, no correlation was found when the results of the healthy group (Group 1) were observed separately, whereas in the group of patients with type 2 diabetes or prediabetes (Group 2) the association was stronger (*r* = −0.59, *P* = 0.027) than in the whole study sample. Similar associations remained after the intervention (*r* = −0.51 and −0.53; *P* = 0.001 and 0.041 for the whole study sample and group 2 respectively).

## Discussion

In the present study, we demonstrated that after only 2 weeks of high‐intensity SIT exercise training and about 48 h after the last training session, the IMCL was increased in the tibialis anterior muscle in both healthy sedentary and diabetic or prediabetic subjects. The correlation between insulin sensitivity (*M*‐value in the hyperinsulinemic euglycemic clamp) and IMCL remained negative throughout the study, and no association was found between the changes in IMCL and insulin sensitivity, even though insulin sensitivity also markedly increased during the intervention, as previously reported (Eskelinen et al. 2015; Sjöros et al. 2018).

We assume that the accumulation of IMCL is due to FFA utilization during exercise and the replenishment and hypercompensation of IMCL after exercise by dietary fat, as has been previously discovered (Décombaz et al. 2001; Larson‐Meyer et al. 2002). Interestingly, the IMCL accumulation was more pronounced after SIT compared to MICT training, even though lipids are generally considered to be used as energy substrates only at low exercise intensities. It is most likely that postexercise energy consumption (EPOC) contributes to the elevated FFA utilization after intense SIT exercise (Trost et al. 1997), thus leading to more substantial IMCL accumulation after SIT than was seen after MICT.

IMCL seems to be very sensitive to the timing of the measurement in relation to the timing of exercise and nutrient consumption. Therefore, direct comparisons between different studies can be questioned. Physical activity and fasting affect IMCL results (Stannard et al. 2002; Machann et al. 2011) as well as lipid infusion (Boden et al. 2001) and fatty diet (Décombaz et al. 2001; Larson‐Meyer et al. 2002). The subjects of this study were instructed not to alter their dietary habits during the intervention. Whereas in interventions with calorie restriction combined with exercise training IMCL has decreased (Tamura et al. 2005) or remained unchanged (Larson‐Meyer et al. 2006) after the intervention.

Aerobic exercise has been found to increase IMCL in the vastus lateralis muscle measured histologically by Oil red O staining (Dubé et al. 2008), MRS (Schrauwen‐Hinderling et al. 2003a), and electron microscopy (Tarnopolsky et al. 2007) and also in the soleus muscle measured by MRS (Haus et al. 2011). Koh and colleagues observed a similar reduction in lipid droplet volume in the subsarcolemmal space after HIIT and moderate‐intensity endurance training in T2D patients, and no change in the intermyofibrillar lipid volume measured by electron microscopy (Koh et al. 2018). In another study, combined strength and endurance training decreased muscle lipid stores in the vastus lateralis muscle measured by MRS, muscle lipid quantified as a single peak (Li et al. 2014). However, it must be acknowledged that correlations between different assessment methods are poor (De et al. 2007). In the TA, any long‐term exercise‐induced changes in IMCL measured by MRS have not previously been evident. In the study of Gan and colleagues post‐exercise measurements were performed 24–36 h after the last bout of exercise and there was no change in IMCL in the TA of overweight male subjects after 6 months of walking/jogging training (Gan et al. 2003). Evidently, different timing of measurements after the training session in different studies makes comparisons between studies somewhat difficult. In our study the measurements were performed about 48 hours after the last training session.

In the present study, training interventions led to similar increases in the IMCL both in the healthy and diabetic or prediabetic subjects. Previously, different responses have also been reported. Nielsen and colleagues found that the increased subsarcolemmal lipid content in diabetic subjects was decreased after 10 weeks of exercise and it approached the level of nondiabetic subjects (Nielsen et al. 2010). Similarly, measured on the following day after finishing 10 days of consecutive exercise training, IMCL was diminished in the group of diabetic subjects, but stayed unaltered in the group of healthy subjects (Bajpeyi et al. 2012). These somewhat contradictory findings can be explained by different timing of muscle samplings and different exercise protocols and study methodology.

The Tibialis anterior was chosen as the site of measurement due to having a uniform muscle fiber orientation that is parallel to the longitudinal axis of the muscle and the magnetic field, and thus a lesser risk for measurement bias (Boesch et al. 1999). However, TA is not the optimal muscle concerning cycling‐based training adaptations as cycling exercise is mainly performed by anterior thigh muscles. Since IMCL has been shown to vary in different muscles (Ortiz‐Nieto et al. 2010), it is not obvious that cycling exercise would alter IMCL in the TA. The TA contributes to cycling effort by dorsiflexing the ankle during pedal upstroke, controlling the ankle joint angle and leveling the pedal. In the present study, the subjects in the SIT group were allowed to stand up on the pedals during the high intensity interval, most likely leading to substantial TA activity, comparable to walking or jogging. Nevertheless, the difference in the activation of TA during cycling compared to walking may also be nonsignificant (Bouillon et al. 2016). Another possible explanation is, that because of its greater intensity, SIT caused a more general overall effect in the whole body. Previously, IMCL has been found to increase immediately after cessation of prolonged exercise in muscles that are less involved in the exercise, probably because of increased FFA availability in the bloodstream (Schrauwen‐Hinderling et al. 2003b). TA muscle is mainly composed of oxidative type I fibers (Johnson et al. 1973). In diabetic and insulin resistant subjects the fiber composition in the skeletal muscles is shifted towards more glycolytic type (Oberbach et al. 2006). Therefore, it is an intriguing speculation that increased IMCL would indicate restored activation of type I fibers.

Current understanding of IMCL in skeletal muscles is that they are benign in nature, even if ectopic lipid accumulation has been previously associated with insulin resistance. Elevated IMCL content has correlated inversely with insulin sensitivity (Goodpaster et al. 1997; Krssak et al. 1999; Nielsen et al. 2010), even if similar correlations have not been found in all studies (Thamer et al. 2003) and, in fact, the relation has also been found to be positive in some studies (Décombaz et al. 2001; Lalia et al. 2016). High IMCL content has been found in diabetic study populations (Petersen et al. 2005; Ingram et al. 2012) as well as in people with the metabolic syndrome (Yokota et al. 2011), but there are also opposite results (Schrauwen‐Hinderling et al. 2007; Szendroedi et al. 2011). On the other hand, also trained endurance athletes have elevated IMCL (van Loon et al. 2004), and if athletes are included in the study, the correlation between IMCL and insulin sensitivity turns positive (Goodpaster et al. 2001). Therefore, it is evident that the relation between IMCL and insulin sensitivity is complex and has different aspects, such as the composition, location, and dynamics of the lipid droplets within the myocytes (Bosma et al. 2012). In the present study, IMCL and insulin sensitivity correlated inversely as well at the baseline as after the intervention; and no correlation was found between the changes of insulin sensitivity and IMCL. The reasons for these varying results are yet to be identified in future studies.

The amount of EMCL has been linked to body adiposity, and in previous studies EMCL has decreased by aerobic exercise training combined with low‐glycemic diet (Haus et al. 2011). We observed a small, but statistically significant decrease in the body adiposity (fat percent) in the present study, but no statistically significant changes in EMCL (*P* = 0.39) and no correlation between changes in body adiposity and EMCL. It should be noted that the training intervention was short and it is possible that a longer intervention is needed to induce a significant effect on EMCL.

This study has some limitations. The training intervention was short and due to technical issues, some of the ^1^H‐MRS measurements were unsuccessful. It is therefore possible that the study was unpowered to fully reveal the power of exercise in modulating the IMCL and EMCL stores and distinguish the differences between the training modalities. The present study is a part of a larger project, therefore the power calculations were made for the main outcome measures, not reported in this article. Additionally, we had both men and women and subjects with variable levels of insulin resistance (T2D or prediabetes), with low number of each in the Group 2, while the Group 1 consisted of healthy male subjects. The sex difference in the groups was accounted for in the statistical analyses by nesting the diabetes factor to the sex factor. However, the amount of IMCL in the TA was not different between men and women in a study with 150 participants at risk for T2D (Machann et al. 2005). Furthermore, we managed to demonstrate a training‐induced accumulation in IMCL after SIT in both healthy and insulin resistant subjects.

In conclusion, in contrast with our hypothesis, training response did not differ between the healthy and diabetic/prediabetic subjects. On the other hand, we found that IMCL increased after SIT compared to MICT, which was in line to our hypothesis. It seems clear that the relation between skeletal muscle insulin sensitivity and intramyocellular lipid is not straightforward, but rather very complex in nature. Additionally, circulating FFAs and nutrition probably have crucial roles in the accumulation of IMCL, and the timing of the measurements must be carefully considered when interpreting the results. Nevertheless, this study confirms the notion that IMCL is not only a malign adaptation to excess lipid flux within the body, nor the reason for skeletal muscle insulin resistance. Instead IMCL serves as an important energy depot during exercise and can be extended by increased energy consumption (that is increased muscle activity) accompanied by sufficient dietary lipid intake. Furthermore, the effects of aerobic exercise on IMCL seem to be similar regardless of insulin sensitivity or the presence of T2D.

## Conflict of Interest

The authors report no conflicts of interest in this work**.**
